# Protective Malaria Vaccine in Mice Based on the *Plasmodium vivax* Circumsporozoite Protein Fused with the Mumps Nucleocapsid Protein

**DOI:** 10.3390/vaccines8020190

**Published:** 2020-04-19

**Authors:** Rodolfo F. Marques, Alba Marina Gimenez, Eduardo Aliprandini, Janaina T. Novais, Diego P. Cury, Ii-Sei Watanabe, Mariana R. Dominguez, Eduardo L. V. Silveira, Rogerio Amino, Irene S. Soares

**Affiliations:** 1 Department of Clinical and Toxicological Analyses, School of Pharmaceutical Sciences, University of São Paulo, São Paulo 05508-000 SP, Brazil; rodolfoferreira@usp.br (R.F.M.); albamarinagimenez@gmail.com (A.M.G.); janaina.novais@usp.br (J.T.N.); maridmz04@gmail.com (M.R.D.); eduardosilveira@usp.br (E.L.V.S.); 2 Center of Cellular and Molecular Therapy, Federal University of São Paulo, São Paulo 04044-010 SP, Brazil; 3 Unit of Malaria Infection & Immunity, Institut Pasteur, 75015 Paris, France; eduardo.aliprandini@pasteur.fr (E.A.); rogerio.amino@pasteur.fr (R.A.); 4 Department of Anatomy, Institute of Biomedical Sciences, University of São Paulo, São Paulo 05508-000 SP, Brazil; diegocury@usp.br (D.P.C.); watanabe@icb.usp.br (I.-S.W.)

**Keywords:** malaria vaccine, *Plasmodium vivax*, circumsporozoite protein, mumps nucleoprotein

## Abstract

*Plasmodium vivax* is the most common species of human malaria parasite found outside Africa, with high endemicity in Asia, Central and South America, and Oceania. Although *Plasmodium falciparum* causes the majority of deaths, *P. vivax* can lead to severe malaria and result in significant morbidity and mortality. The development of a protective vaccine will be a major step toward malaria elimination. Recently, a formulation containing the three allelic variants of the *P. vivax* circumsporozoite protein (PvCSP—All epitopes) showed partial protection in mice after a challenge with the hybrid *Plasmodium berghei* (Pb) sporozoite, in which the PbCSP central repeats were replaced by the VK210 PvCSP repeats (Pb/Pv sporozoite). In the present study, the chimeric PvCSP allelic variants (VK210, VK247, and *P. vivax*-like) were fused with the mumps virus nucleocapsid protein in the absence (NLP-CSP_R_) or presence of the conserved C-terminal (CT) domain of PvCSP (NLP-CSP_CT_). To elicit stronger humoral and cellular responses, *Pichia pastoris* yeast was used to assemble them as nucleocapsid-like particles (NLPs). Mice were immunized with each recombinant protein adjuvanted with Poly (I:C) and presented a high frequency of antigen-specific antibody-secreting cells (ASCs) on days 5 and 30, respectively, in the spleen and bone marrow. Moreover, high IgG titers against all PvCSP variants were detected in the sera. Later, these immunized mice with NLP-CSP_CT_ were challenged with Pb/Pv sporozoites. Sterile protection was observed in 30% of the challenged mice. Therefore, this vaccine formulation use has the potential to be a good candidate for the development of a universal vaccine against *P. vivax* malaria.

## 1. Introduction

*Plasmodium vivax* is the second most prevalent species causing malaria in the world, occurring mainly in South and Southeast Asia, the Western Pacific, the Eastern Mediterranean, Central America, and South America [1]. Recent data estimate that 7.5 million malaria cases are caused by *P. vivax* annually in the world, and nearly 2.5 billion people are living in areas at risk of this infection [1]. The mortality induced by *P. vivax* infection is considered to be very low when compared to *P. falciparum* infection. However, *P. vivax*-derived morbidity has significantly increased during the last few years due to the development of chloroquine resistance by the parasite [2]. In addition, several countries have reported an increase in the number of malaria cases with serious complications including anemia, respiratory distress syndrome, cerebral malaria, and malnutrition [3]. Although public health experts have considered the development of a malaria vaccine to be a research priority [4], clinical research for *P. vivax* vaccines has been mostly neglected. In fact, only a few trials for vaccines against *P. vivax* malaria have been performed [5].

The circumsporozoite protein (CSP) is the most prominent antigen on the surface of *Plasmodium* sporozoites. The most advanced malaria vaccine candidate, Mosquirix^TM^ (RTS,S), was developed based on the conserved C-terminal domain and the central repeats domain of the *P. falciparum* CSP combined with the hepatitis B virus surface antigen (HBsAg). Its expression occurs in the virus-like particle (VLP) assemble that is conjugated with AS01E adjuvant [6]. In phase III clinical trials, the RTS,S vaccination resulted in a 30%–50% reduction in disease incidence and a 26%–35% prevention of cerebral malaria, though the duration of this efficacy was seen to be limited [7]. Immunological studies carried out 12 months post-RTS,S immunization demonstrated that protection against the first or recurrent malaria events was related to the generated anti-CSP IgG antibodies, which targeted repeat and C-terminal domains [8].

*P. vivax* CSP (PvCSP) comprises a central domain of tandem repeat sequences flanked by two non-repetitive conserved sequences, the N- and C-terminal domains. Three different variants of the central domain of PvCSP are described: VK210, VK247, and *P. vivax*-like [9,10,11]. These three variants present worldwide distribution and can be identified by both serological and molecular methods [12,13,14,15]. Antibodies directed against the PvCSP central repeats domain can neutralize the sporozoite infectivity, allowing for the acquisition of sterile protection [16]. A VLP formulation containing PvCSP-based antigens (VK210 and VK247) combined with HBsAg showed promising results both alone and in combination with other antigens [17,18]. However, other studies have demonstrated the importance of the three alleles in conferring worldwide protection against *P. vivax* malaria based on the newly developed CSP-based vaccine [19]. 

The only vaccine formulation against *P. vivax* malaria tested in phase I/IIa clinical trials so far, VMP001/AS01B, contains the repeat sequences of VK210 (nine repeats) and VK247 (one repeat) fused to N- and C-terminal conserved regions of PvCSP. This recombinant protein has shown the ability to induce a potent immune response in malaria-naïve, healthy volunteers. This vaccine does not induce sterile protection against *P. vivax* in any of the volunteers. However, 59% of the vaccinees presented a significant time delay in the development of parasitemia as compared to the control group [20].

In addition to the conserved domains, our research group has developed recombinant vaccine formulations targeting all three variants of *P. vivax* CSP capable of eliciting immune responses in mice in the last few years [21]. These constructs have been expressed in both prokaryotic [21] and eukaryotic (yeast) systems [22,23]. The recombinant protein PvCSP-All_CT_ has been found to be highly immunogenic in mice when administered with Poly (I:C) adjuvant. When challenged with a transgenic parasite (Pb/PvVK210), 4/6 mice demonstrated protective immune responses. In comparison to the controls, the immunized group displayed a 20-fold reduction in the liver parasite burden [22].

VLPs are known for their intrinsic "self-assembling" capability and their ability to induce a much stronger stimulation of B- and T-cell-mediated immune responses [23]. Therefore, the use of VLPs in vaccine formulations has been extensively studied. Although these molecular structures resemble whole virus particles, they do not have genetic material and are therefore unable to infect cells and self-replicate, making them a very safe choice for vaccine formulations [24]. Additionally, VLPs are known for their efficiency at presenting heterologous target antigens—a strategy that can radically alter the magnitude of the immune response, thereby significantly improving the protection that the new vaccines confer [25,26,27]. 

The VLP advantages are widely known despite technical difficulties such as yield, purification, and storage of these particles for the development of new vaccines [28]. Memory immune response against the viral particle is another concerning matter when considering VLPs as vaccine platforms [29]. 

Therefore, our study aimed to overcome these issues by fusing the vaccine antigens to a core viral protein rather than the surface proteins (characteristic of VLPs). For this, we used the mumps viral nucleocapsid protein (NP) in our formulation due to its ability to form stable nucleocapsid-like particles (NLPs) when expressed by the *P. pastoris* expression system [30]. In addition, this protein showed low seroprevalence in mumps-vaccinated individuals [31]. 

In this study, NLPs were formed by fusing two chimeric proteins containing the PvCSP sequences to the mumps-NP sequence as a vaccine candidate to elicit stronger immune responses in terms of magnitude and breadth. This strategy provided enhanced protection in mice against a malarial challenge with a transgenic Pb/PvVK210 parasite. 

## 2. Materials and Methods

### 2.1. Generation of Recombinant NLP-Fused Proteins 

The gene sequences encoding recombinant PvCSP proteins NLP-CSP_CT_ and NLP-CSP_R_ were synthesized using codon optimization for expression in *P. pastoris* (GenScript, Piscataway, NJ, USA). Both proteins contained the three variant tandem repeats domains of PvCSP [22] fused with the mumps virus nucleocapsid protein (UniProtKB/Swiss-Prot: AFO62160) sequence. The NLP-CSP_CT_ protein also contained the conserved C-terminal of PvCSP following the repeats domains. The mumps virus NP sequence was associated with the sequences of PvCSP using an amino acid linker (GAAGAGA) [32]. The sequences described above were cloned into the restriction sites *Eco*RI and *Not*I of the plasmid pPIC9K (Invitrogen, Life Technologies Corporation USA Inc., Waltham, MA, USA). In accordance with a previous study, this enabled the secretion and expression of the proteins under control of the alcohol oxidase gene promoter (AOX1). Additionally, both recombinant proteins contained a hexa-histidine tag at the C-terminus end, enabling further purification by Nickel Resin Affinity Chromatography. To avoid N-glycosylation, the sequences were analyzed with the NetNGlyc 1.0 server (DTU Health Tech, Technical University of Denmark) and three putative N-linked glycosylation (NxS/T) sites found were replaced by the following amino acids (NxS/T to AxS/T).

### 2.2. Transformation of P. pastoris Host Strains 

The *P. pastoris* strain GS115 (his4) (Invitrogen, Life Technologies Corporation USA Inc., Waltham, MA, USA) was transformed by electroporation using linearized plasmids treated with *Sal* I (New England Biolabs Inc., Ipswich, MA, USA). The transformed yeast was then grown on histidine-deficient media for the selection of the His^+^ clones. According to the instructions of the *Pichia* Expression Kit from Invitrogen, the selection of clones with multiple inserts was carried out on YPD (Yeast extract – Peptone – Dextrose) plates containing geneticin (Sigma-Aldrich, St. Louis, MO, USA) (from 0.25 to 4.0 mg/mL).

### 2.3. Production of PvCSP 

The selected clones were grown for 24 h at 30 °C with constant stirring (230 rpm) in 40–200 mL of medium containing 1% glycerol (BMGY) to enable expression of the recombinant proteins. The cells were then harvested by centrifugation, solubilized in 40–200 mL of medium containing 1% methanol (BMMY), and cultured at 28 °C with constant stirring (230 rpm). Induction was maintained by the daily addition of 1% methanol throughout the incubation period of 72–96 h. The cells were harvested by centrifugation, and the supernatant was filtered out using 0.45 μm membranes (Merck Millipore, Billerica, MA, USA). 

### 2.4. Purification of Recombinant Proteins 

The purification of the recombinant proteins was performed in a two-step procedure (affinity and ion exchange chromatographies). Initially, the supernatant containing the solubilized protein was subjected to affinity chromatography using a HisTrap™ FF nickel (GE Healthcare USA Inc., Pittsburgh, PA, USA) column coupled to the FPLC ÄKTA prime plus (GE Healthcare USA Inc., Pittsburgh, PA, USA) system. Elution occurred against an imidazole gradient (15–400 mM, USB, Affymetrix USA Inc., Santa Clara, CA, USA) in a phosphate–chloride buffer (20 mM NaH_2_PO_4_ (Synth, Labsynth Products for Laboratories BRA Ltda., Diadema, Brazil) 20 mM Na_2_HPO_4_ (Synth, Labsynth Products for Laboratories BRA Ltda.), and 0.5 M NaCl (Synth, Labsynth Products for Laboratories BRA Ltda.), pH = 6.0).

Fractions containing the recombinant proteins, identified on 12% SDS-PAGE gels stained with Coomassie blue solution, were dialyzed on 10,000 MWCO SnakeSkin Pleated Dialysis Tubing membrane (Thermo Fisher Scientific USA Inc., Waltham, MA, USA) against 20 mM Tris-HCl, (pH = 8.0). After dialysis, the proteins were filtered (0.45 μm) and subjected to a purification step by ion exchange chromatography using the HiTrap™ QFF Column (GE Healthcare USA Inc.), also coupled to the ÄKTA system. Protein elution occurred in a linear gradient from 0 to 1 M NaCl over 20 mM Tris-HCl buffer (Invitrogen, Life Technologies Corporation USA Inc., Waltham, MA, USA). Fractions containing the chimeric recombinant proteins were then dialyzed against phosphate-buffered saline (PBS) (8 mM NaH_2_PO_4_, 2.3 mM Na_2_HPO_4_, 130 mM NaCl, pH = 7.4) overnight, with constant stirring at 4 °C.

Protein concentration was determined by densitometry analysis using ImageQuant™ TL version 8.1 software (GE Healthcare USA Inc.) and compared to a calibration curve with defined concentrations of bovine serum albumin (BSA, Invitrogen, Life Technologies Corporation USA Inc.).

### 2.5. Transmission Electron Microscopy 

The purified proteins NLP-CSP_CT_ and NLP-CSP_R_ were diluted to the concentration of 2 μg/mL and placed in Formvar/carbon-coated electron microscopy grids (EMS # FCFT200-Cu). The grids were covered and incubated for 20 min in a dry environment. The lower grids were washed for 2 min in distilled water, then in 1% glutaraldehyde (Sigma-Aldrich # G5882) for 5 min and again in water for 2 min. These steps were repeated 7 times. Then, the grids were placed in 50 μL of 4% uranyl acetate (EMS, # 22400) for 7 min. The grids were examined the next day under a transmission electron microscope, Tecnai FEI G20 200 kV, at the Microscopy Center in the Department of Development and Cell Biology at the Biomedical Sciences Institute of the University of São Paulo, under the supervision of Professor Ii-Sei Watanabe.

### 2.6. Immunoblot Assay 

The recombinant proteins NLP-CSP_CT_ and NLP-CSP_R_ were electrophoresed using 12% SDS-PAGE gel and, thereafter, electrotransferred onto Hybond N nitrocellulose membranes (GE Healthcare USA Inc.). For the protein transfer, a transfer buffer (160 mM glycine, 25 mM Tris, and 20% (v/v) methanol) was used, and the reaction occurred at 90 V for 30 min using the Bio-Rad semi-wet transfer system (Bio-Rad Laboratories USA Inc., Hercules, CA, USA). The membranes were stained with Ponceau-S solution (0.1% Ponceau red (Bio-Rad Laboratories USA Inc.) and 10% acetic acid) and then incubated for 16–18 h at 4 °C with a blocking solution (5% skimmed milk (Molico®, Nestlé S.A., Vevey, VD, Switzerland), 2.5% (w/v) bovine serum albumin (Sigma-Aldrich, St. Louis, MO, USA) in PBS). The membranes were then incubated for 1 h at room temperature (RT) with an individual primary antibody (anti-PvCSP-VK210 (2F2), anti-PvCSP-VK247 (2E10.E9) or anti-mumps-NP (7B10) (Abcam; Ab9880) monoclonal antibodies (mAbs)) at a dilution of 1:2000 (v/v) and a polyclonal anti-*P. vivax*-like antibody [22] at a dilution of 1:2000 (v/v). After 3 washings of 10 min each with PBS-T ((0.05% Tween 20 (v/v), Invitrogen, Life Technologies Corporation USA Inc.), the membrane was incubated with a peroxidase-conjugated anti-mouse IgG antibody (KPL, Kirkegaard & Perry Laboratories USA Inc., Gaithersburg, MA, USA) diluted 1:3000 in blocking solution for 1 h at RT. After 3 washing steps with PBS-T, the membrane was developed using the SuperSignal kit (Thermo Fisher)

### 2.7. High-Performance Reverse-Phase Liquid Chromatography (RP-HPLC) 

The purified recombinant NLP-CSP_CT_ and NLP-CSP_R_ proteins were analyzed through high-performance reversed-phase liquid chromatography (RP-HPLC) using a Vydac C4 column (4.6 mm × 250 mm, for particles of 300 Hm) in a Shimadzu LC Solution HPLC system (Shimadzu JPN Corp., Kyoto, KY, USA). The technique was performed at the Laboratory of Toxins and Natural Products of Algae (School of Pharmaceutical Sciences, University of São Paulo). A 05–100% acetonitrile (Merck KGaA DEU Inc.) gradient comprising 90% acetonitrile with 0.1% trifluoroacetic acid (TFA, Thermo Fisher Scientific USA Inc.) was used at approximately 24 °C under a flow of 1 mL/min for 40 min. The elution was monitored with a UV-Visible Absorbance Detector (Shimadzu SPD M20A, Shimadzu JPN Corp., Kyoto, Japan) at 214 nm.

### 2.8. Circular Dichroism 

The circular dichroism (CD) tests were carried out on the JASCO-J810 spectropolarimeter (Jasco ITA Corp., Milano, MI, Italy) equipped with a continuous temperature control system (Central Analytical Laboratory, School of Pharmaceutical Sciences, University of São Paulo). The recombinant NLP-CSP_CT_ and NLP-CSP_R_ proteins were loaded in a 5 mm quartz cuvette and underwent 8 UV scans at a speed of 20 nm/min. These scans were performed at 260–200 nm with bandwidth/response time being 1 nm/1 second at 20 °C. The spectra were corrected by subtracting the buffer signal (PBS), while the molar ellipticity (θ) was calculated for the prediction of the secondary structures of the recombinant proteins using the CDNN Secondary Structure Analysis software (Applied Photophysics GBR Ltd., Leatherhead, Su, UK).

### 2.9. Mice Immunization 

Six- to eight-week-old female C57BL/6 mice were purchased from the animal facility of the School of Pharmaceutical Sciences/Chemistry Institute (University of Sao Paulo). All animal experiments were approved by the Animal Care and Use Committee of the University of São Paulo (CEUA/FCF 74.2016-P531). Briefly, mice were subcutaneously (s.c.) immunized 3 times with the vaccine formulation of recombinant protein/adjuvant. For each dose, a final volume of 100 µL (10 µg of protein/sterile PBS/50 μg of Poly (I:C) HMW (Polyinosinic-polycytidylic acid - High Molecular Weight) (Invitrogen, Life Technologies Corporation USA Inc., Waltham, MA, USA) was injected over the flank per mouse.

### 2.10. Antibody Measurement 

For immunological assays, 14 days after each immunization, blood was collected from the submandibular vein (*n* = 6 mice per group). Sera were analyzed for the presence of antibodies against each PvCSP variant as well as N- and C-terminal regions of the PvCSP. Antibodies were detected through an enzyme-linked immunosorbent assay (ELISA), as previously described [21]. The recombinant proteins FliC-VK210, FliC-VK247, and FliC-*P. vivax*-like [33] were used as target antigens (200 ng/well). Following overnight incubation at RT, the plates were washed with a solution of PBS 0.05% Tween-20 (PBS-T) and blocked with a blocking solution (PBS, 5% (w/v) skimmed milk) for 2 h at 37 °C. Serial dilutions starting with 1:200 of murine polyclonal sera were added to the wells and incubated for 1 h at RT. After a washing step with PBS-T, peroxidase-labeled goat anti-mouse IgG (Sigma, St. Louis, MO, USA) was added to each well at a 1:3000 dilution. The OPD/acid stop system was then used to determine anti-PvCSP titers based on the highest dilution of sera yielding an A_492_ value higher than 0.1 [22]. 

The EUROIMMUN anti-mumps-virus ELISA (IgG) assays (EI 2630–9601 G) were used to determine the mumps antibody seroprevalence. Positive and negative controls along with calibrators for semi-quantitative calculation and sample preparation was used according to the manufacturer’s instructions. The cut-off values for the anti-mumps-virus ELISA and NP ELISA were as follows: negative < 0.30; borderline ≥ 0.30–0.42; positive ≥ 0.42. The recombinant protein Mumps-NP (Abcam; ab74560) was used as the target antigen (200 ng/well) in an ELISA performed to determine the titers of the mumps virus nucleoprotein-specific antibodies. We assayed serum samples collected from 80 individuals living in different malaria-endemic areas in the north of Brazil (40 *P. vivax*-infected and 40 non-infected) [34]. We also assayed 12 samples from individuals who were immunized or had mumps living in non-endemic areas. 

### 2.11. Antibody Secreting B-Cell Measurement by ELISPOT 

For antibody-secreting cell (ASCs) measurement, a second group of mice was immunized as described above (*n* = 4 mice per group). We performed the ELISPOT assays as previously described by Fabris et al. [35]. Briefly, ELISPOT plates (Millipore; MSHAN4B50) were coated with 1 μg/mL of recombinant proteins overnight at 4 °C to identify PvCSP-specific ASCs. Plates were washed 4 times with PBS 0.05% Tween 20 (PBS-T) and 4 times with PBS and blocked for 2 h at 37 °C with RPMI medium supplemented with 10% FBS (R10 medium). Splenocytes and bone marrow cells were isolated, counted, and plated in serial 3-fold dilutions in R10 medium. ELISPOT plates were incubated overnight in a 5% CO_2_ incubator at 37 °C. Plates were washed 4 times with PBS and 4 times with PBS-T, followed by incubation with anti-mouse IgG-biotin conjugated antibodies (BD), diluted 1:1000 in PBS-0.05% Tween 20 containing 2% FBS solution (PBS-T-F) for 90 min at RT. Wells were again washed 4 times with PBS-T before the addition of Avidin D-HRP (KPL) diluted 1:3000 in PBS-T-F. After a 3-h incubation at RT, plates were washed 4 times with PBS-T and 4 times with PBS. Spots were developed with filtered 3-amino 9-ethylcarbazole (AEC) substrate as recommended by the manufacturer (BD). To stop the reaction, wells were washed with running water and plates were dried before spot counting. Images of spots were defined by an automated stereomicroscope (KS ELISPOT, Zeiss, Oberkochem, Germany).

### 2.12. Parasites, Mice, and Mosquitoes 

Sporozoites (spz) from *Plasmodium berghei* ANKA expressing *P. vivax* CSP VK210 repeats (Pb/PvVK210) were obtained as previously described [36]. C57BL/6JRj mice were purchased from Janvier Labs. All animal experiments were approved by the Animal Care and Use Committee of Institut Pasteur (CETEA Institut Pasteur 2013-0093, Ministère de l’Enseignement Supérieur et de la Recherche MESR 01324) and were performed in accordance with European guidelines and regulations (directive 2010/63/EU). For all tests, 6- to 8-week-old females were used and allocated randomly to cages. Two independent immunization/blind challenge experiments were performed using 7 animals per experiment, as described previously [37]. 

*Anopheles stephensi* mosquitoes (SDA500 strain) were reared at the Centre for Production and Infection of *Anopheles* (CEPIA) at the Institut Pasteur using standard procedures. For the production of rodent *Plasmodium* spp. spzs, mosquitoes were fed on infected RjOrl:SWISS mice 1-2 days after emergence and kept in a humidified chamber at 21 °C. One week after infection, Pb/PvVK210-infected mosquitoes were fed on naïve RjOrl:SWISS mice. For footpad injections, Pb/PvVK210 spzs were collected from mosquito-infected salivary glands 21–28 days after the infectious blood meal [37].

### 2.13. Murine Sporozoite Challenge 

Transgenic Pb/PvVK210 spz were maintained in female *A. stephensi* mosquitoes. The total number of spz was determined using a Kova glass slide; 5000 spz/μL of PBS were microinjected in the footpad skin using a 35–36 g needle with a NanoFil syringe (World Precision Instruments, Sarasota, FL, USA) in control and immunized mice [37]. Parasitemia was determined by flow cytometry, performed during days 4–10 after the spz challenge. For this, 200,000 erythrocytes were examined for each sample. A quantitative analysis of protection was performed using the parasitemia log values on day 5 post-infection, when the blood parasites were still exponentially growing [37].

### 2.14. Statistical Analyses 

One-way analysis of variance (ANOVA) and Tukey’s honestly significant difference (HSD) test were used to compare the results from different groups. Differences were considered statistically significant when *p* < 0.05. In the challenge experiments, the significant protection was determined using Fisher’s exact test.

### 2.15. Data Availability

The data that support the findings of this study are available from the corresponding author upon reasonable request.

## 3. Results

### 3.1. Generation, purification, and recognition by mAbs of the proteins NLP-CSP_CT_ and NLP-CSP_R_

**Figure 1:**
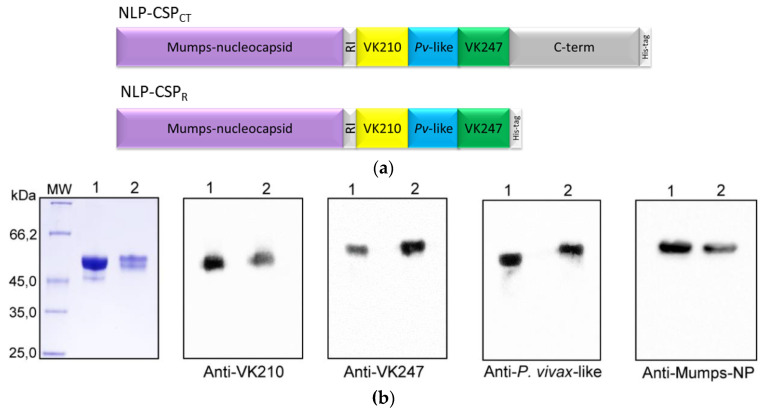

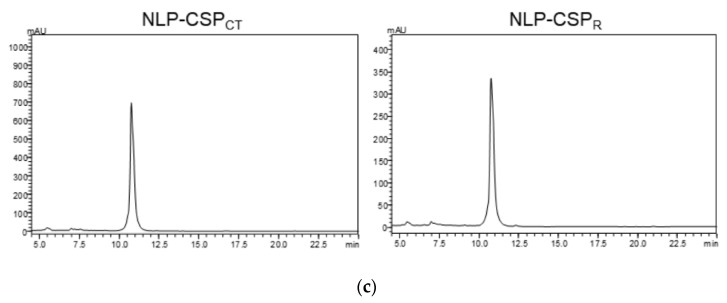
Generation, purification, and MAb recognition of nucleocapsid-like particles (NLP)-recombinant proteins. (**a**) Schematic representation of *P. vivax* circumsporozoite (PvCSP) proteins fused with mumps virus nucleocapsid expressed from *P. pastoris*. (**b**) NLP-CSP_CT_ and NLP-CSP_R_ are represented as lines 1 and 2, respectively. SDS-PAGE analysis under reducing conditions of purified recombinant proteins gel-stained with Coomassie blue (1 µg of protein per lane) and protein recognition by mAbs anti-VK210, anti-VK247, and anti-NP, and by polyclonal anti-*P. vivax*-like antibody. (**c**) The purity of the proteins after chromatography was analyzed by RP-HPLC, in which the gradient elution was developed by combining 0.1% trifluoroacetic acid (TFA) in water and 0.1% TFA in 90% acetonitrile at 24 °C, 1 mL/min for 40 min in a C18 column.

We started our study by designing the NLP-CSP_CT_ and NLP-CSP_R_ constructs. Codon-optimized genes were synthesized containing the same coding regions for the Mumps-NP, 7 amino acid linker (GAAGAGA), and hexa-histidine tag for both constructs. However, their PvCSP sequences were distinct. While the NLP-CSP_CT_ protein had the Mumps-NP sequence, linker, and PvCSP-All_CT_ sequences (RI region from N-terminal domain, representative sequences of the three amino acid repeat variants and the C-terminal domain of the PvCSP protein [22] and his-tag), the NLP-CSP_R_ protein comprised the Mumps-NP sequence, linker, PvCSP RI region, and repeat variants (Figure 1a). Recombinant proteins were produced and purified as described and separated by SDS-PAGE under reducing conditions (Figure 1b). 

To determine whether the produced polypeptides retained the epitopes recognized by specific mAbs generated against radiation-attenuated *P. vivax* sporozoites, we performed an immunoblot analysis. As depicted in Figure 1b, purified proteins were recognized by specific mAbs generated against radiation-attenuated *P. vivax* sporozoites (mAb VK210 2F2 and mAb VK247 2E10.E9), as well as polyclonal anti-*P. vivax*-like antibodies [22] and anti-mumps nucleoprotein antibody (mAb 7B10). After chromatographic separation, the purity of each protein was analyzed by RP-HPLC. Chromatograms revealed a high degree of purity for both recombinant proteins (Figure 1c).

### 3.2. Preliminary Structural Characterization of the Proteins NLP-CSP_CT_ and NLP-CSP_R_

**Figure 2:**
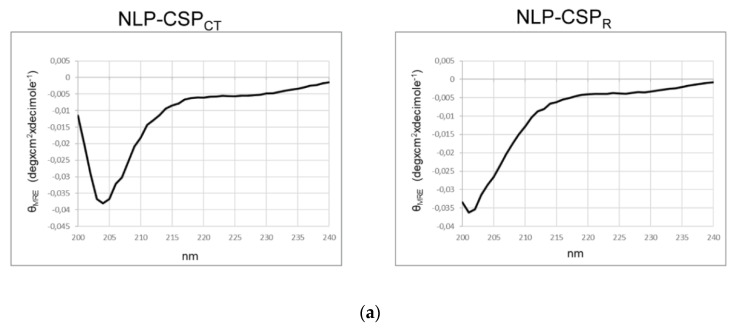

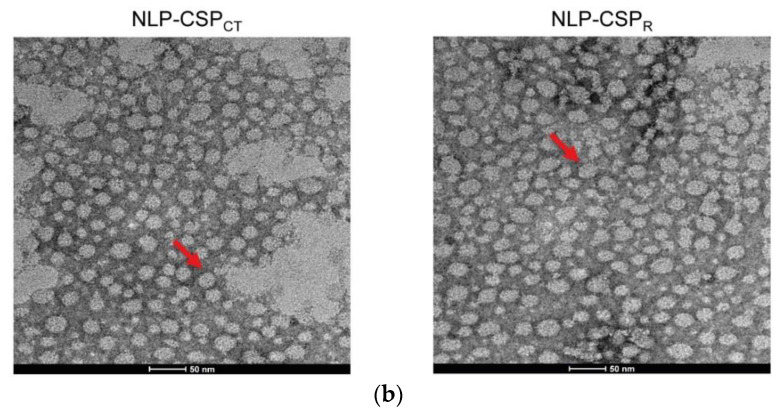
Structural characterization of NLP-recombinant proteins. (**a**) The secondary structure of NLP-CSP_CT_ and NLP-CSP_R_ was analyzed by circular dichroism (CD) spectrum. The CD spectrum of the recombinant proteins was recorded from 190 to 260 nm using a JASCO-J815 spectropolarimeter. (**b**) The NLP-CSP_CT_ and NLP-CSP_R_ proteins were negatively stained with 0.5% uranyl acetate and visualized by Transmission Electron Microscopy (TEM).

The secondary structures of the recombinant proteins were analyzed using far-UV CD spectroscopy. The CD spectra of the two proteins (Figure 2a) showed negative bands near 222 and 208 nm, consistent with random coiled proteins.

The following estimated percentages of α-helix, β-sheet, and β-turn for each protein were predicted: (i) NLP-CSP_CT_: 12.4% α-helix, 20.7% anti-parallel β-sheet, 19.5% parallel β-sheet, 22.4% β-turn; (ii) NLP-CSP_R_: 12.4% α-helix, 20.7% anti-parallel β-sheet, 19.6% parallel β-sheet, 22.5% β-turn. However, the differences in the spectra patterns near 200 nm indicated a higher stability of NLP-CSP_CT_. 

The electron microscopy (EM) examination of purified NLP proteins revealed large quantities of circular structures, which displayed some features common to VLPs (Figure 2b). These circular structures appeared as flexible forms 15–20 nm in diameter. Formation of NLPs in yeast expression systems occurred in the absence of other viral proteins, as previously described [30]. 

### 3.3. Seroprevalence of Antibodies Specific to the Mumps Virus and Its Nucleoprotein in Residents of Malaria-Endemic and Non-Endemic Areas 

**Figure 3:**
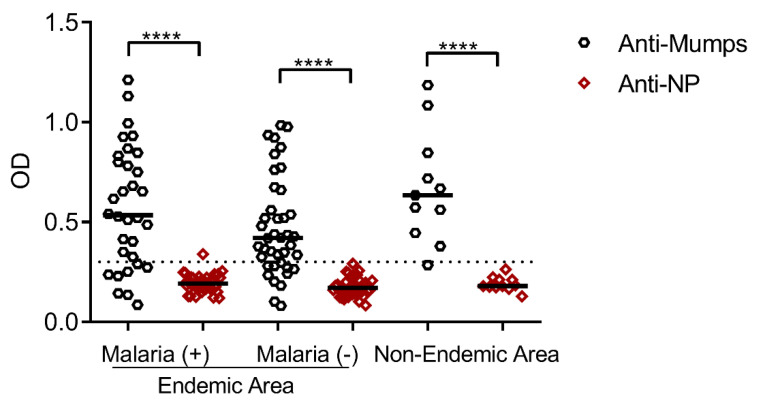
Comparison of antibody response against the mumps virus and nucleocapsid proteins (NPs). The serology of *P. vivax*-infected individuals (*n* = 40), non-infected individuals (*n* = 40), and individuals who were immunized or had mumps from a non-endemic *P. vivax* area (*n* = 12) was investigated using a commercial ELISA kit. The dashed line in the chart indicates the minimum threshold (OD = 0.3) for considering an individual to be immunized against the mumps virus, as indicated in the supplier’s manual. The results were statistically compared using one-way ANOVA followed by Tukey’s test. Significant differences between groups are denoted on the graph: * *p* < 0.05, ** *p* < 0.01, *** *p* < 0.001, and **** *p* < 0.0001. Non-significant (ns) differences are indicated (*p* > 0.05).

Before the further assessment of NLP-CSP_CT_ immunogenicity, the seroprevalence of antibodies specific to the mumps virus was investigated. Considering the presence of the mumps viral antigen in the described malaria vaccine formulations, this analysis is necessary. Any immunological memory against the mumps virus in the general population (generated by vaccination or natural exposure) could interfere with the immune response against PvCSP as demonstrated with other formulations [29]. Therefore, we measured the serum antibodies specific to the mumps virus and its NP in individuals from malaria-endemic and non-endemic areas. While most of the subjects tested mumps-positive, demonstrating that most of them had been vaccinated against mumps, the subjects were negative for Mumps-NP (*p* < 0.0001) regardless of the area of origin or any previous exposure to malaria (Figure 3). This result is in agreement with the low seroprevalence of antibodies specific to Mumps-NP reported previously [31].

### 3.4. Antibody Responses in Mice Vaccinated with NLP-CSP_CT_ and NLP-CSP_R_

**Figure 4:**
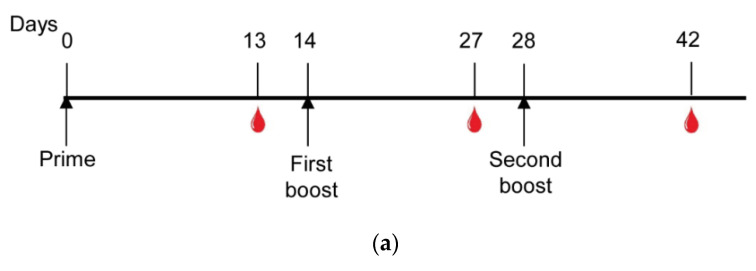

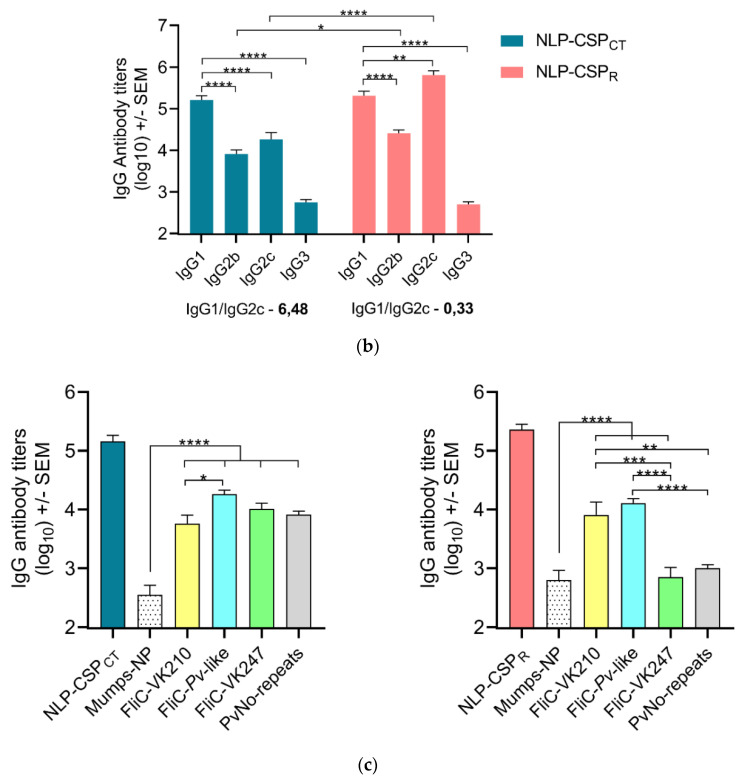
Specific antibody response in mice immunized with NLPs. (**a**) Mice immunization schedule: Groups of six C57BL/6 mice were immunized s.c. with three doses 14 days apart with NLPs (10 µg/dose) in the presence of the adjuvant Poly (I:C). The antibody response was then analyzed according to the timeline described. (**b**) Antibody subclasses against each NLP were analyzed, and the IgG1/IgG2c ratios are expressed below the graph. (**c**) The elicited Abs specifically recognized the central repeats of the three allelic variants, as shown by the assays for the repeats fused to flagellin FliC (FliC-PvCSP-repeats) or N-C-terminal regions (FliC-No repeats) and Mumps-NP after three doses. All results are expressed as the arithmetic mean titers of each group (log_10_ ± SEM) and were statistically compared using one-way ANOVA followed by Tukey’s test. Significant differences between groups are denoted on the graph: * *p* < 0.05, ** *p* < 0.01, *** *p* < 0.001, and **** *p* < 0.0001. Non-significant (ns) differences are indicated (*p* > 0.05).

The immunogenicity of the recombinant proteins was assessed after their in vivo administration in conjugation with Poly (I:C), a synthetic analog of double-stranded RNA (dsRNA) agonist of TLR3 [38] in mice. Each animal received three doses of each protein 14 days apart from each other in a homologous prime-boost vaccination regimen (Figure 4a). When the titers of PvCSP-specific IgG subclasses were evaluated in these groups on day 14 after the second boost, the responses differed significantly. Responses against the NLP-CSP_CT_ protein resulted in predominately IgG1 rather than IgG2c (ratio–6.48), while a balanced ratio between IgG1 and IgG2c was found against NLP-CSP_R_ (ratio: 0.33). When comparing antibody titers of the same subclass, but in the different groups, we observed a significant increase between the IgG2b (*p* = 0.0279) and IgG2c (*p* < 0.0001) (Figure 4b). In addition, we evaluated antibody specificity against the homologous proteins that contain the repeat regions of each PvCSP protein variant (FliC’s) [33], Mumps-NP protein, the NLP-CSP_R_ and NLP-CSP_CT_ chimeric proteins, and a protein containing the PvCSP non-repeat regions alone (Pv-No repeat) (Figure 4c). 

High-antibody titers were measured against homologous proteins (higher than 10^5^) and each repeat variant, in both groups (higher than 10^4^), except for NLP-CSP_R_-vaccinated mice, in which antibody titers for the VK247 variant were significantly lower in comparison to other repeat variants (*p* < 0.0001 when compared to *P. vivax*-like and *p* = 0.0001 to VK210). The serum from NLP-CSP_CT_-vaccinated mice presented antibody titers for *P. vivax*-like variant that were significantly higher than the VK210 (*p* < 0.0395), but not significant for VK247. Furthermore, mice immunized with NLP-CSP_CT_ presented antibody titers for the no-repeat variants that were as high as the antibody titers for the repeat variants. Mice vaccinated with NLP-CSP_R_ presented low antibody titers for no-repeat variants (Figure 4c). As expected, the antibody titer against Mumps-NP protein was significantly lower in comparison to almost all variants in both groups, except for VK247 of the NLP-CSP_R_ group (*p* = 0.9998). These data led to the conclusion that both vaccine formulations induced poor immune responses to Mumps-NP. However, only the vaccine formulation containing NLP-CSP_CT_ induced high antibody titers to all PvCSP repeat variants and conserved domains. These data and the greater stability of NLP-CSP_CT_ shown by CD spectra analyses encouraged the further study of this recombinant protein.

### 3.5. Detection of NLP-CSP_CT_-Specific ASCs in Spleen and Bone Marrow of Vaccinated Mice

**Figure 5:**
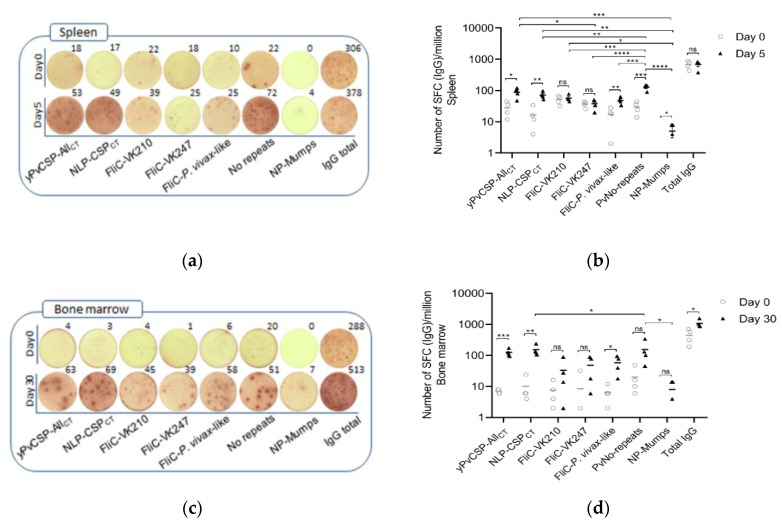

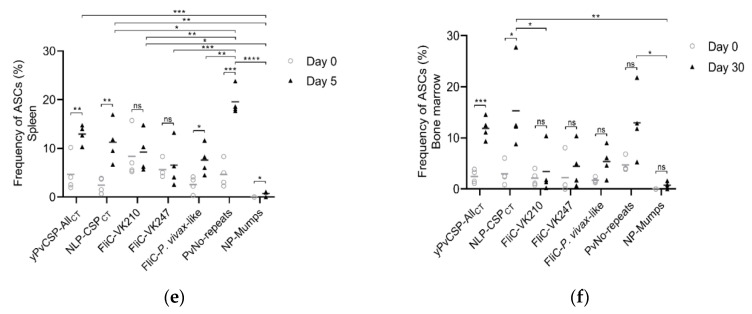
Antibody-secreting cell (ASC) response in NLP-CSP_CT_-immunized mice. Groups of four C57BL/6 mice were immunized with three doses of NLP-CSP_CT_ conjugated with Poly (I:C) adjuvant. The antigen-specific ASC response in the spleen was measured on days 0 and 5, and in the bone marrow on days 0 and 30 after the last dose. A representative example of the ASC response specific to yPvCSP-All_CT_, NLP-CSP_CT_, FliC-VK210, FliC-VK247, and FliC-*P. vivax*-like in the spleen is shown in (**a**), while that of the bone marrow is shown in (**c**). The wells shown in the figure were plated with 0.5 × 10^5^ cells. The total ASCs were measured for IgG secreting cells specific to the above-mentioned proteins. The data points represent the average ASC counts observed per million cells, while the bars represent the ± SEM (**b** and **d**) and frequency (**e** and **f**). The results were statistically compared using one-way ANOVA followed by Tukey’s test. Significant differences between groups are denoted on the graph: * *p* < 0.05, ** *p* < 0.01, *** *p* < 0.001, and **** *p* < 0.0001. Non-significant (ns) differences are indicated (*p* > 0.05). The experiment was performed twice with four mice per group.

Because we observed high antibody titers to all PvCSP repeat variants in mice immunized with NLP-CSP_CT_, we performed an ELISPOT assay to evaluate NLP-CSP_CT_-specific antibody-secreting cell (ASC) generation in the spleen and bone marrow of immunized mice at days 5 and 30, respectively, after the second boost. More specifically, the ELISPOTs were performed against the two chimeric proteins PvCSP-All_CT_ and NLP-CSP_CT_, each of the three PvCSP repeat alleles and no-repeat regions, Mumps-NP, and total IgG-secreting cells. We measured ASC frequency in splenocytes 5 days after the second boost, as this is usually the time when their frequency reaches the peak after immunizations [39]. An increased number of ASCs specific to *P. vivax*-like repeats (*p* = 0.0097), the Pv-No repeats (*p* = 0.0401) portion of the CSP, yPvCSP-All_CT_ (*p* = 0.0139)_,_ NLP-CSP_CT_ (*p* = 0.0032), and Mumps-NP (*p* = 0.0006) was observed in the spleens of immunized mice (Figure 5a,b). However, the number of ASCs specific to VK210 and VK247 was not significantly different from days 0 to 5, nor was the frequency of total IgG secreting cells. By contrast, bone marrow cells were tested for ASCs only 30 days after the second boost, as they take longer to migrate to that location [40]. An augmented frequency of ASCs specific to the two chimeric proteins PvCSP-All_CT_ and NLP-CSP_CT_ was found in the bone marrow (Figure 5c,d). Although the third dose increased the overall response to the two chimeric proteins, yPvCSP-All_CT_ (*p* = 0.0004) and NLP-CSP_CT_ (*p* = 0.0031), to *P. vivax*-like repeats (*p* = 0.0278) and total IgG (*p* = 0.0207), no increase in expression was observed in each protein region individually: VK210, VK247, Pv-No repeats, and Mumps-NP. The non-specific responses (total IgG ASCs) were, as expected, the same in both organs regardless of the time after stimuli. The CSP-specific responses accounted for 10% of the total ASCs in the spleen and bone marrow on days 5 and 30 post-stimuli, respectively (Figure 5e,f).

Therefore, the ELISPOT data correlate with the high antibody titers observed by ELISA in the serum of these mice. The magnitude of the antibody titers presented in the serum was the same as the antibodies secreted by long-lived ASCs present in the bone marrow and newly formed ASCs (briefly present in recirculation and in the secondary lymphoid organs where they are formed, as well as in inflamed tissues for as long as they are inflamed). Because NLP-CSP_CT_ immunization induces high antibody titers against all PvCSP repeat and non-repeat regions, it may represent the large amount of ASCs generated after the first and second doses that reached the bone marrow. Some ASCs are seen to be formed after the third dose, reflecting in the increased number of ASCs against some portions of the protein seen in the spleen 5 days after the third immunization and in the antibody titers against *P. vivax*-like, as well as in the increased number of ASCs in the bone marrow 30 days after the third immunization to the whole vaccine protein. 

### 3.6. Assessment of Vaccine Efficacy

**Figure 6:**
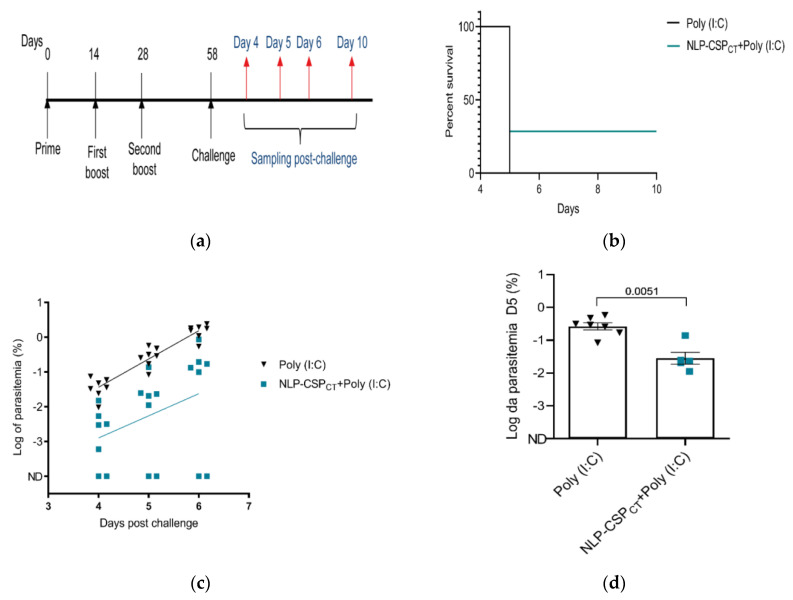
Evaluation of parasitemia after challenge in mice immunized with a prime-boost regimen. (**a**) C57BL/6 mice were immunized s.c. with Poly (I:C) or a mixture of Poly (I:C) and 10 µg of recombinant protein, NLP-CSP_CT_. On day 58 after priming, the mice were challenged with 5000 Pb/PvVK210 transgenic sporozoites. (**b**) Surveillance curve with the percentage of non-infected mice in each experimental group is shown. (**c**) Parasitemia was analyzed by flow cytometry. The percentage of infected red blood cells (iRBCs) on days 4, 5, and 6 post-infection (p.i.) was expressed as log values for normalization before statistical analysis. (**d**) The log of parasitemia on day 5 (D5) post-challenge was measured in mice in each of the immunized groups. Data from two independent experiments and significance were determined by two-tailed unpaired t-test (Mann–Whitney test).

One of the most effective ways to analyze vaccine efficacy is through a challenge test. This approach has been used previously and has proven effective at demonstrating vaccine-induced protection [21,22,41]. However, *P. vivax* does not infect rodents. Considering that *P. berghei* is a murine-infecting parasite, we used the chimeric parasite Pb/PvVK210, in which the repeats of the PbCSP were replaced by the PvCSP (VK210), as previously described [36]. Thus, mice were immunized 3 times with NLP-CSP_CT_ + Poly (I:C) or only the adjuvant. Thirty days later, mice were challenged with 5000 Pb/PvVK210 sporozoites (Figure 6a). Sterile protection was observed in 2 out of 7 mice until day 10 post-challenge, whereas all control mice were infected by day 4 post-challenge (Figure 6b). In addition to the partial sterilizing protection, a ~10-fold decrease in parasitemia on day 5 in the mice immunized with NLP-CSP_CT_ + Poly I:C was observed in comparison to the controls (Figure 6c, *p* = 0.0012). Moreover, the decrease in parasitemia was still significant in the 5 immunized unprotected mice compared to controls (Figure 6d, *p* = 0.0051).

## 4. Discussion

The use of viral particles as antigen delivery systems has been shown to be an excellent strategy in the development of more efficient vaccines. Some successful cases of commercially available VLP-based vaccines are for Human Papilloma Virus (HPV), such as Cervarix^®^, Gardasil^®^ & Gardasil9^®^, and for Hepatitis B Virus (HBV) [42].

We have previously shown that the PvCSP expressed in *P. pastoris* was highly immunogenic, eliciting antibodies capable of recognizing *P. vivax* sporozoites and conferring moderate protection against a challenge test [22]. The need to develop a new strategy for improving the efficacy of a *P. vivax* vaccine led to the exploration of the mumps virus NP as an antigen carrier [43]. This carrier was selected after taking into account two facts: the internal protein is weakly recognized even in mumps-vaccinated individuals [31], and the *P. pastoris* secreted expression system was already successfully used for the expression of mumps NLPs [30]. This is described as one of the most powerful systems for recombinant protein expression, yielding proteins with high purity [44] and the ability to express viral particles in the form of VLPs [45,46]. Using the same strategy, we successfully expressed, characterized, and purified two mumps NLPs carrying PvCSP sequences that remained stable and presented the appropriate structural conformation, as evidenced by the mean diameter of the particle corresponding to mumps nucleocapsid [30], confirmed after scanning electron microscopy.

The importance of inducing high levels of PvCSP-specific antibodies was highlighted in a study comparing the immunogenicity of VMP001 with CSV-S,S, which is an RTS,S-like vaccine candidate containing hepatitis B surface antigen (HBsAg) and a protein fusing the VMP001 sequences to HBsAg [47]. Although CSV-S,S induced higher levels of PvCSP repeat variants specific antibodies than did soluble VMP001, there is a major concern about the immune responses generated against the viral components. By contrast, the mumps-NP particle present in our formulations proved to be an excellent vaccine candidate as emphasized by the low seroprevalence of the antibodies against mumps-vaccinated/exposed individuals or individuals living in *P. vivax* endemic areas [31], thereby avoiding the possible interference of memory cells against the viral particle. The lack of a PvCSP N-terminal domain containing CD4 T cell epitopes in our formulations could present a further advantage compared to VMP001, as CD4^+^ T cell responses were seen to be directed to the N-terminal region in 90% of individuals vaccinated with VMP001; this response was not protective against controlled human malaria infection [20]. 

The two vaccine formulations tested in this study induced high antibody titers to all three PvCSP repeat regions, being dominated by the *P. vivax*-like variant among them, in murine sera 14 days after three immunization doses. Regarding the adjuvant selected to these immunizations, Poly (I:C), it activates different pattern-recognition receptors, such as TLR3, RIG-I/MDA5, and PKR, triggering multiple inflammatory pathways, including NF-kB and IRF [48,49]. By eliciting an inflammatory Th1-like response in mice and humans, it was hypothesized that such a response could contribute to protection [50]. Previously, our research group showed that Poly (I:C) was responsible to enhance not only the immunogenicity of PvCSP recombinant proteins upon vaccination [21,33] but also protection after a parasitic challenge [22,41]. In this study, the NLP-CSP_R_ vaccine formulation induced a predominance of IgG2c over IgG1. In an animal model of *P. falciparum* infection, a vaccine candidate also induced anti-CSP IgG2 antibodies which were associated with protection [51]. On the other hand, the NLP-CSP_CT_ protein displayed the opposite pattern, with predominance of IgG1. Thus, our data suggest that the presence of the C-terminal region of the PvCSP in NLP-CSP_CT_ protein may alter the antigen conformation, spotlighting other epitopes related to an IgG2 response. Even so, it provided protection with decreased parasitemia and enhanced survival of immunized and challenged mice. However, it remains elusive whether a vaccine formulation with such particular responses in mice could present a similar pattern in humans and still be protective.

The high level of antibody titers in the serum is a result of the increased number of specific ASCs in the spleens of these mice 5 days after the second boost. Most of the ASCs generated after the first and second doses of the vaccine should have reached the bone marrow by day 0 following the second boost, indicating that the “basal” level of specific ASCs could already be high. In our vaccine formulation, once all three repeat variants are presented in the same chimeric protein at the same molecular ratio, differences in the kinetics of ASCs specific to *P. vivax*-like and no-repeats are not expected. Although the mechanism involved is not within the scope of this paper, some of the following hypotheses can be taken into consideration: (i) Some protein regions may be more available to antibody binding than others in the tertiary protein structure, which would confer an advantage for B-cell activation. This is one of the most important features of B-cell responses and has been extensively studied for antigens such as HA from the influenza virus and gp120 from HIV [52,53]. While the preference of the antibody response to recognize the repeat portions of the CSP during infections has also been documented, our chimeric construct combines all repeat variants into one protein and seems to prioritize the B-cell immune response to individual components that remain ambiguous [47,54]; (ii) Furthermore, the recognition of some epitopes can interfere with the availability of the antibody binding to the other epitopes. Although this a rare phenomenon, it has been documented in the antibody immunity to PfCSP in infected patients [55]. 

Disregarding the mechanism involved, it is undeniable that the third immunization elicited de novo ASC formation to PvCSP non-repeat regions and the *P. vivax*-like variant. Not all of the ASCs formed at this time point will contribute to the overall long-term immunity observed in the bone marrow, but some will and can be detected when the overall immunity is analyzed (ASC specific to PvCSP-All_CT_ and NLP-CSP_CT_). This increase in overall long-term immunity indicates the need for a third immunization dose and may reflect on the vaccine efficacy. It is interesting to note that the same ASC response is observed against PvCSP-All_CT_ and NLP-CSP_CT_, thereby indicating that the fusion of NP with respect to the chimeric CSP protein construct did not affect the recognition of the PvCSP epitopes. Furthermore, when the different protein components were analyzed individually, no increase in the number of ASCs in the bone marrow 30 days after the third dose was observed. This suggests that efficient long-term immunity was already formed by the first and second immunization doses (serological imprinting). Hence, the third immunization dose seems to enhance the already-existing long-term immunity, being of particular importance to strengthening the immunity required to achieve memory cells that contribute to the overall protection, as is seen for the influenza vaccine [56].

Our formulation was able to confer sterile protection in approximately 30% of the animals, in addition to a significant decrease (approximately 10-fold) in the parasitemia of non-sterile protected animals. In a brief comparison with our previous results [22], although the methodology used to assess the protection was the same, there were significant differences in some points. To use an immunization route more suitable for comparison with future results in human vaccination, the i.p. route used in Gimenez et al. was changed to the s.c. route in our study. Challenge conditions were also strengthened from 4000 to 5000 sporozoites, while the parasitemia evaluation was highly improved from smear to flow cytometry, which is a more sensitive and precise methodology. For these reasons, we consider that the partial sterile protection observed in this study is at least equivalent to the previous results. Regarding other formulations that were able to confer a higher level of protection in murine models, such as Rv21 [17], the comparison is even harder: not only is the immunization route different (i.m. vs. s.c.), but transgenic parasites were also constructed using different approaches [17,36] and the challenge systems vary from i.v. to s.c., respectively.

In cases of malaria caused by *P. vivax*, a complicating factor in the pathology of the disease is the presence of the latent form of the parasite, the hypnozoite, which is responsible for the relapses of the disease. In this regard, the 10-fold decrease in parasitemia goes beyond what is seen, as some of the sporozoites that were not able to reach the liver gave rise to hypnozoite forms. On the other hand, we were not able to test this possibility of hypnozoite production after the parasitic challenge as the transgenic Pb/Pv parasite is still biologically *P. berghei* and does not form hypnozoites in mice. Thus, the partial protection provided by this vaccine candidate may be related to multiple possibilities: (i) different folding of the vaccine antigen in comparison to the native parasitic protein, hiding or changing important epitopes related to protection; (ii) reduced T cell responses, critical for antibody responses; (iii) higher pathogenicity of the transgenic parasite used in the mouse challenge due to the increased amount of sporozoites inoculated and/or route of inoculation; etc. Extrapolating these data to humans, the partial protection could hypothetically contribute to a decrease in the number of relapses [57].

The number of sporozoites (spz) used in the infection model and the use of transgenic parasites (Pb/Pv) are the two other points to be considered. For the challenge test, 5000 spz were used, which is a very high number, as in a natural infection by a mosquito bite, an individual is inoculated with less than 40 spz [58]. Although malarial transgenic parasites are an important tool for elucidating vaccine efficacy, their use may produce results that underestimate vaccine efficacy. This transgenic parasite is constructed by replacing only the repeated region of the *P. berghei* CS protein with the corresponding amino acids of the VK210 variant of *P. vivax* [36]. However, the vaccine formulation in this study elicited antibodies against repeats of VK210, VK247, and *P. vivax*-like variants, RI and C-terminal region. The challenge test indicated only the protective efficacy of antibodies directed to the repeats of the VK210 variant. Therefore, the efficacy of the vaccine formulation developed in this study may be even greater when considered in case of a natural infection.

## 5. Conclusions

In summary, we have constructed, expressed, and purified a chimeric vaccine with a high purity level, comprising the mumps virus nucleoprotein sequence in fusion with different PvCSP allelic variants along with its C-terminal region. This vaccine formulation was able to generate high titers of specific antibodies that promoted moderate protection in infected animals. A future scope of this study would entail a detailed understanding of the mechanism by which this formulation confers partial protection. This, in turn, shall improve its efficacy for potential application as a new *P. vivax* vaccine.
